# Prevalence and Clinical Significance of Herpesvirus Infection in Populations of Australian Marsupials

**DOI:** 10.1371/journal.pone.0133807

**Published:** 2015-07-29

**Authors:** Kathryn Stalder, Paola K. Vaz, James R. Gilkerson, Rupert Baker, Pam Whiteley, Nino Ficorilli, Liliana Tatarczuch, Timothy Portas, Kim Skogvold, Garry A. Anderson, Joanne M. Devlin

**Affiliations:** 1 The Faculty of Veterinary and Agricultural Sciences, The University of Melbourne, Melbourne, Victoria, Australia; 2 Australian Wildlife Health Centre, Healesville Sanctuary, Healesville, Victoria, Australia; 3 Veterinary and Research Centre, Tidbinbilla Nature Reserve, Via Tharwa, Australian Capital Territory, Australia; 4 Conservation Medicine Program, School of Veterinary and Life Sciences, Murdoch University, Perth, Western Australia, Australia; 5 Perth Zoo Veterinary Department, Perth Zoo, South Perth, Western Australia, Australia; University of Liverpool, UNITED KINGDOM

## Abstract

Herpesviruses have been reported in several marsupial species, but molecular classification has been limited to four herpesviruses in macropodids, a gammaherpesvirus in two antechinus species (*Antechinus flavipes* and *Antechinus agilis*), a gammaherpesvirus in a potoroid, the eastern bettong (*Bettongia gaimardi*) and two gammaherpesviruses in koalas (*Phascolarctos cinereus*). In this study we examined a range of Australian marsupials for the presence of herpesviruses using molecular and serological techniques, and also assessed risk factors associated with herpesvirus infection. Our study population included 99 koalas (*Phascolarctos cinereus*), 96 eastern grey kangaroos (*Macropus giganteus*), 50 Tasmanian devils (*Sarcophilus harrisii*) and 33 common wombats (*Vombatus ursinius*). In total, six novel herpesviruses (one alphaherpesvirus and five gammaherpesviruses) were identified in various host species. The overall prevalence of detection of herpesvirus DNA in our study population was 27.2% (95% confidence interval (CI) of 22.6–32.2%), but this varied between species and reached as high as 45.4% (95% CI 28.1–63.7%) in common wombats. Serum antibodies to two closely related macropodid herpesviruses (macropodid herpesvirus 1 and 2) were detected in 44.3% (95% CI 33.1–55.9%) of animals tested. This also varied between species and was as high as 92% (95% CI 74.0–99.0%) in eastern grey kangaroos. A number of epidemiological variables were identified as positive predictors for the presence of herpesvirus DNA in the marsupial samples evaluated. The most striking association was observed in koalas, where the presence of *Chlamydia pecorum* DNA was strongly associated with the presence of herpesvirus DNA (Odds Ratio = 60, 95% CI 12.1–297.8). Our results demonstrate the common presence of herpesviruses in Australian marsupials and provide directions for future research.

## Introduction

Herpesviruses are enveloped, double stranded DNA viruses that have been identified in species across the animal kingdom, including vertebrate and invertebrate species. Extensive coevolution of herpesviruses with their host species is thought to be largely responsible for their exceptional adaptation to their natural hosts, and plays an important role in their survival strategy [1,2]. Herpesviruses are well known for their capacity to induce lifelong infections. Herpesvirus infections are characterised by a primary infection event, with or without acute disease, followed by variable periods of subclinical latency, with subsequent episodes of virus reactivation and shedding during periods of stress or immune-compromise. It is this biological strategy that contributes significantly to the survival and dissemination success of herpesviruses in their host species [2].

Herpesviruses were first identified in Australian marsupials in 1975 when an outbreak of disease and sudden death in a group of captive parma wallabies (*Macropus parma*) led to the isolation of what is now known as Macropodid herpesvirus 1 (MaHV-1) from the renal tissue of an affected animal [3]. Affected wallabies exhibited various clinical and pathological abnormalities; including rhinitis, conjunctivitis, pneumonia, cloacal ulceration, and variable splenic, pulmonic and hepatic necrosis. Intranuclear inclusion bodies were occasionally identified [3]. Since then, further outbreaks of disease in various macropod species have led to the discovery of three additional herpesvirus species. Macropodid herpesvirus 2 (MaHV-2), an alphaherpesvirus similar but distinct from MaHV-1 [4,5] was detected in samples from grey dorcopsis wallabies (*Dorcopsis luctuosa*) and a quokka (*Setonix brachyurus*). Macropodid herpesvirus 3 (MaHV-3), a gammaherpesvirus, was identified in captive and free-living eastern grey kangaroos (*Macropus giganteus*) [6,7]; and recently Macropodid herpesvirus 4 (MaHV-4), an alphaherpesvirus associated with respiratory and possibly neurological disease was detected in a free-living eastern grey kangaroo [8]. A wide range of Australian marsupials (up to 23%) were found to have virus neutralising antibodies against MaHV-1 in an early seroprevalence study, with higher prevalence and antibody titres found to be associated with captivity and advancing age [9]. More recently a gammaherpesvirus, denoted Potoroid herpesvirus 1 (PotHV-1), was identified in four free-living eastern bettongs (*Bettongia gaimardi*) as part of a comprehensive health surveillance program during translocation [10]. Two gammaherpesviruses have also been recently identified in koalas (*Phascolarctos cinereus*) [11,12] and a novel gammaherpesvirus species was found in a yellow-footed antechinus (*Antechinus flavipes*) and an agile antechinus (*Antechinus agilis*) [13]. Whilst herpesvirus particles have been detected by electron microscopy in a common wombat (*Vombatus ursinus*) [14], molecular detection and classification of wombat herpesviruses have not previously been reported.

Many Australian marsupial species are now considered vulnerable and threats to the survival of populations include habitat destruction through urbanisation or fire, introduced predators and competitors, inbreeding and disease. These threats have been better characterised for some species than others, and the risk and consequences of herpesvirus infections in these populations remains to be quantified. Whilst most herpesvirus outbreaks reported in Australian marsupials have occurred within captive environments [3,15–17], the identification of MaHV-3 in a group of free-ranging eastern grey kangaroos exhibiting respiratory disease [7] and the isolation of MaHV-4 from a free-living eastern grey kangaroos with respiratory disease [8], indicates that some herpesviruses may have the potential to negatively impact free-living marsupial populations.

This study aimed to examine the prevalence of herpesviruses in samples collected from captive and free-ranging Australian marsupials using molecular and serological techniques. This study also aimed to identify any risk factors or signs of disease associated with herpesvirus infection in these animals. The marsupial populations targeted included koalas, eastern grey kangaroos *(Macropus giganteus)* and common wombats. Samples from other marsupial species were also included opportunistically.

## Materials and Methods

### Sample collection

Approval for this study (Animal Ethics ID 1112058.1) was granted from the Animal Ethics Committee for the Faculty of Veterinary Science, The University of Melbourne. In 2011, samples were collected from free-living macropods, common wombats and koalas that presented to the Australian Wildlife Health Centre, Healesville Sanctuary, Healesville, Victoria (37.682° S, 145.532° E) and other local wildlife centres in Victoria as a result of trauma, disease or abandonment. Sterile cotton swabs (Copan Italia) were used to collect swab samples from the conjunctivae, nasal cavity, oropharynx and cloaca from each animal and the prepuce of male animals. Blood samples were also collected from each animal where possible, and the serum stored at -20°C. Swab samples were stored in 500 μl Dulbecco’s minimal essential medium (DMEM, Sigma-Aldrich) supplemented with 1% v/v foetal bovine serum (Sigma-Aldrich), 10 mM HEPES, pH 7.6 and 50 μg/ml gentamicin (Sigma-Aldrich) at –70°C. Demographic and clinical data including species, sex, age, weight, location found, presence or absence of pouch young, body condition, clinical signs observed and concurrent diseases were recorded for each animal and entered into an electronic database (Microsoft Access, 2010). Swab samples and corresponding animal health data were also opportunistically gathered from other Australian marsupial species during their assessment for other purposes. This included captive and free-living Tasmanian devils from Healesville Sanctuary and Tasmania, respectively. Furthermore, swab samples and health data from an additional 68 free-ranging Victorian koalas collected in 2010 during a previous investigation into *Chlamydia* infection [18] were included in our study. The study population is summarised in Table 1.

**Table 1:** Overview of the population of Australian marsupials sampled for this study during 2010 and 2011, and results from the PCR detection of herpesvirus DNA in the collected swab samples.

Family	Species	Scientific name	Captive	Free-living	Total	PCR positive	Prevalence % (95% CI)
Macropodidae	Eastern grey kangaroo	*Macropus giganteus*	0	96	96	24/96	25.0 (16.7–34.9)
	Swamp wallaby	*Wallabia bicolor*	0	15	15	4/15	26.7 (7.8–55.1)
	Brush-tailed rock wallaby	*Petrogale penicillata*	10	3	13	0/13	0.0 (0.0–20.6)
	Tammar wallaby	*Macropus eugenii*	8	0	8	0/8	0.0 (0.0–31.2)
	Yellow-footed rock wallaby	*Petrogale xanthopus*	3	0	3	0/3	0.0 (0.0–63.2)
	Red-necked wallaby	*Macropus rufogriseus*	1	0	1	0/1	0.0 (0.0–95.0)
Potoroidae	Long-nosed potoroo	*Potorous tridactylus*	9	0	9	0/9	0.0 (0.0–28.3)
Phascolarctidae	Koala	*Phascolarctos cinereus*	10	89	99	33/99	33.3 (24.2–43.5)
Dasyuridae	Tasmanian devil	*Sarcophilus harrisii*	29	21	50	17/50	34.0 (21.2–48.8)
	Eastern quoll	*Dasyurus viverrinus*	1	1	2	0/2	0.0 (0.0–77.6)
Vombatidae	Common wombat	*Vombatus ursinus*	0	33	33	15/33	45.5 (28.1–63.7)
Peramelidae	Southern brown bandicoot	*Isoodon obesulus*	0	11	11	1/11	9.1 (0.23–41.3)
	Eastern barred bandicoot	*Perameles gunnii*	3	0	3	0/3	0.0 (0.0–63.2)
Phalangeridae	Common brushtail possum	*Trichosurus vulpecula*	0	2	2	0/2	0.0 (0.0–77.6)
Pseudocheiridae	Common ringtail possum	*Pseudocheirus peregrinus*	0	1	1	0/1	0.0 (0.0–95.0)
All families	All species		74	272	346	94/346	27.2% (22.6–32.2)

### Molecular investigations

DNA was extracted from 200 μl of each swab sample using VX Universal Liquid Sample DNA Extraction Kits (Qiagen) and a Corbett X-tractor Gene Robot (Corbett Robotics). Negative extraction controls utilised sterile phosphate buffered saline (PBS) only. Positive extraction controls utilised supernatant from cell cultures infected with the avian alphaherpesvirus, infectious laryngotracheitis virus. Extracted DNA was then used as a template in a nested pan-herpes PCR, using primers targeting a conserved region of the herpesvirus DNA polymerase gene [19]. PCR negative controls containing no DNA template were also included. PCR products underwent DNA purification (QIAquick Gel Extraction Kit, Qiagen) and sequencing (Big Dye Terminator version 3.1, Applied Biosystems). Sequences were compared with published nucleotide sequences in the GenBank database (NCBI, 2013) using the BLAST-N online algorithm. Further refinement and analysis of the sequence data was performed with Geneious Pro 5.1.6 (Biomatters Ltd) software. The predicted amino acid sequences of the detected herpesviruses were aligned with representative members from the three *Herpesviridae* subfamilies from a range of host species, and an unrooted maximum-likelihood phylogenetic tree was generated from this sequence alignment using the Jones–Taylor–Thornton model of amino acid replacement, as described previously [12].

### Cell culture and virus isolation

Selected swab samples that were positive for the presence of novel herpesviruses, or herpesviruses that had not been isolated previously, were used to inoculate wallaby fibroblast cells [20] or primary wombat kidney cell cultures in a closed culture system and incubated at 35–37°C. The primary wombat kidney cells were generated from kidney tissue harvested from a wombat joey using standard trypsin disaggregation techniques. Inoculated cells were evaluated daily for evidence of cytopathic effect (CPE) consistent with herpesvirus infection. Cell cultures were passaged as necessary. The presence of herpesvirus virions was confirmed using electron microscopy.

### Serological analysis

Serum-virus neutralisation assays were used to detect antibodies to the closely related alphaherpesviruses MaHV-1 and MaHV-2 in serum samples (n = 79) collected from animals from eight different marsupial species. Serum samples were thawed at room temperature before centrifugation to remove residual cellular debris. Samples were then heat inactivated at 56°C for 30 min before testing as previously described [8]. Briefly, serum samples were diluted 1:2 in sterile media and added to 100 times the median tissue culture infective dose (100 TCID_50_) of MaHV-1 or MaHV-2. Serum samples that prevented viral CPE in wallaby fibroblast cells were recorded as positive for the presence of virus-neutralising antibodies. A serum sample that had previously tested positive in our laboratory using these methods was used as a positive control. Other control wells were cell-only controls, and no-serum (cell and virus only) controls. Virus titrations were performed to confirm the viruses were used at the correct dilution. Antibody levels were not determined by further dilution of positive serum samples.

### Statistical analysis

Statistical analysis was performed using Minitab 16.1.0 statistical software (Minitab Inc., 2010). Binary logistic regression was utilised to evaluate the significance of epidemiologic factors in the probability of detecting herpesvirus DNA in a swab sample from an individual animal. Analyses were performed at a species level for host species where 10 or more infected individuals were present in the study population, and also for all marsupial species combined. The analysis of all marsupial species combined included data from the individual animals reported in this study (Table 1) in addition to data from 27 woylies (*Bettongia penicillata*) and 24 eastern bettongs which were originally tested as part of this project [10,21] but the results of which have been analysed at a species level separately as a component of studies focussed on those animals. Univariable analysis was initially performed for each variable under investigation, and variables were assessed for collinearity using contingency tables. Reference levels were selected on the basis of greater sample size and biological sense. Inclusion in the preliminary final model was based on a log likelihood p value ≤ 0.25 and absence of collinearity with other included variables. A backward elimination method was used to determine the final multivariable logistic regression model and eliminated variables were individually returned to the model to assess confounding (considered to be > 25% change in the coefficient of retained significant variables). A two-tailed p ≤ 0.05 was considered to be statistically significant.

## Results

### Molecular investigations

**Fig 1:**
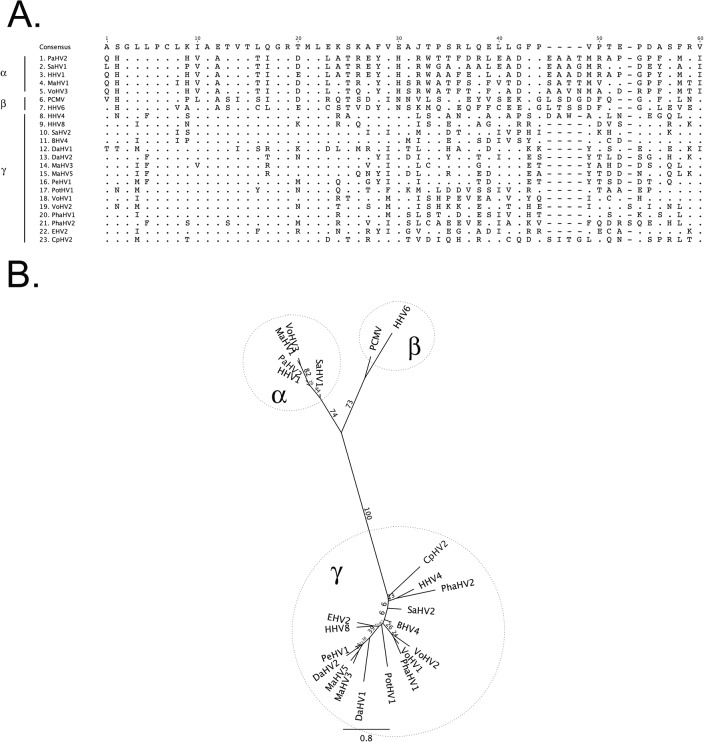
Predicted amino acid alignment and phylogenetic tree of the novel marsupial herpesviruses. A: Alignment of the predicted amino acid sequence of a portion of the DNA polymerase gene of the novel marsupial herpesviruses, along with other herpesviruses from the three herpesvirus sub-families. B: Maximum likelihood tree generated from the alignment. Bootstrap values of 100 replicates are displayed on the tree branches. Novel herpesvirus species are underlined. Key: PaHV-2 = papiine herpesvirus 2 (AAN87165.1); SaHV-1 = saimiriine herpesvirus 1 (YP_003933809.1); HHV-1 = human herpesvirus 1 (NP_044632.1); MaHV-1 = macropodid herpesvirus 1 ([22]); VoHV-3 = vombatid herpesvirus 3 (novel sequence); PCMV = porcine cytomegalovirus (AF268042.1); HHV-6 = human herpesvirus 6A (NP_042931.1); HHV-4 = human herpesvirus 4 (YP_401712.1); HHV-8 = human herpesvirus 8 (ACY00400.1); SaHV-2 = saimiriine herpesvirus 2 (NP_040211.1); BHV-4 = bovine herpesvirus 4 (NP_076501.1); DaHV-1 = dasyurid herpesvirus 1 [13]; DaHV-2 = dasyurid herpesvirus 2 (novel sequence); MaHV-3 = macropodid herpesvirus 3 (ABO61861.1); MaHV-5 = macropodid herpesvirus 5 (novel sequence). PeHV-1 = peramelid herpesvirus 1 (novel sequence); PotHV-1 = potoroid herpesvirus 1 [10]; VoHV-1 = vombatid herpesvirus 1 (novel sequence); VoHV-2 = vombatid herpesvirus 2 (novel sequence); PhaHV-1 = phascolarctid herpesvirus 1 (AEX15649.1); PhaHV-2 = phascolarctid herpesvirus 2 (AFN66528.1); EHV-2 = equine herpesvirus 2 (NP_042605.1); CpHV-2 = caprine herpesvirus 2 (ADV92276.1).

The prevalence of herpesvirus DNA detection by PCR in the marsupial species evaluated ranged from 0.0–45.5%, with an overall prevalence of 27.2% (Table 1). Nucleotide sequence analyses and comparison with sequences in the GenBank database identified six novel herpesviruses; three in wombats, one in swamp wallabies, one in Tasmanian devils and one in a southern brown bandicoot. Using current herpesvirus nomenclature these novel herpesviruses were putatively designated Vombatid herpesvirus 1–3 (VoHV1-3), Macropodid herpesvirus 5 (MaHV-5), Dasyurid herpesvirus 2 (DaHV-2) and Peramelid herpesvirus 1 (PeHV-1), respectively. Five of the six novel viruses were gammaherpesviruses, with one alphaherpesvirus (VoHV-3) detected in wombats. Sequence data and phylogenetic analysis for the novel herpesvirus species are presented in Fig 1.

Each of the novel herpesviruses was restricted to a single host species. In wombats, VoHV-1, VoHV-2 and VoHV-3 were detected in 5/33 (15%), 7/33 (21%) and 3/33 (9%) of the sample population, respectively. In one wombat both VoHV-1 and VoHV-2 were detected. In swamp wallabies, Tasmanian devils and southern brown bandicoots, the novel herpesviruses (MaHV-5, DaHV-2 and PeHV-1, respectively) were detected in 4/15 (27%), 17/50 (34%) and 1/11 (9%) of animals, respectively.

In addition to the six novel herpesviruses identified in this study, nucleotide sequence analyses also revealed the presence of four previously described herpesviruses in the samples collected from the animals listed in Table 1. Macropodid herpesviruses 3 and 4 were detected in 19/96 (20%) and 5/96 (5%) of eastern grey kangaroos, respectively. Phascolarctid herpesviruses 1 and 2 were detected in 10/99 (10%) and 23/99 (23%) of koalas, respectively. In one koala, both PhaHV-1 and PhaHV-2 were detected. Sequence data was unavailable for herpesvirus DNA detected in an additional koala.

Herpesvirus DNA was detected in swabs collected from a number of different anatomical sites, with some variations between species (Table 2).

**Table 2:** Anatomical sites of herpesvirus DNA detection in swab samples collected from Australian marsupials in 2010 and 2011.

		Herpesvirus positive swabs per site (%) ^b^				
Family	Species ^a^	Conjunctiva	Nostril	Oropharynx	Cloaca	Prepuce
Macropodidae	Eastern grey kangaroo	9/60 (15)	12/90 (13)	13/42 (31)	9/87 (10)	4/24 (17)
Phascolarctidae	Koala	10/97 (10)	7/27 (26)	5/12 (42)	19/87 (22)	2/6 (33)
Dasyuridae	Tasmanian devil	1/29 (3)	3/29 (10)	14/50 (28)	7/29 (24)	4/13 (31)
Vombatidae	Common wombat	3/27 (11)	9/33 (27)	3/24 (13)	2/33 (6)	1/9 (11)

^a^ Only species that were sampled in relatively large numbers, from multiple anatomical sites, are included.

^b^ Herpesvirus DNA was sometimes detected in more than one swab from the same animal, swabs were not collected from every anatomical site from every animal.

### Cell culture, virus isolation and electron microscopy

**Fig 2:**
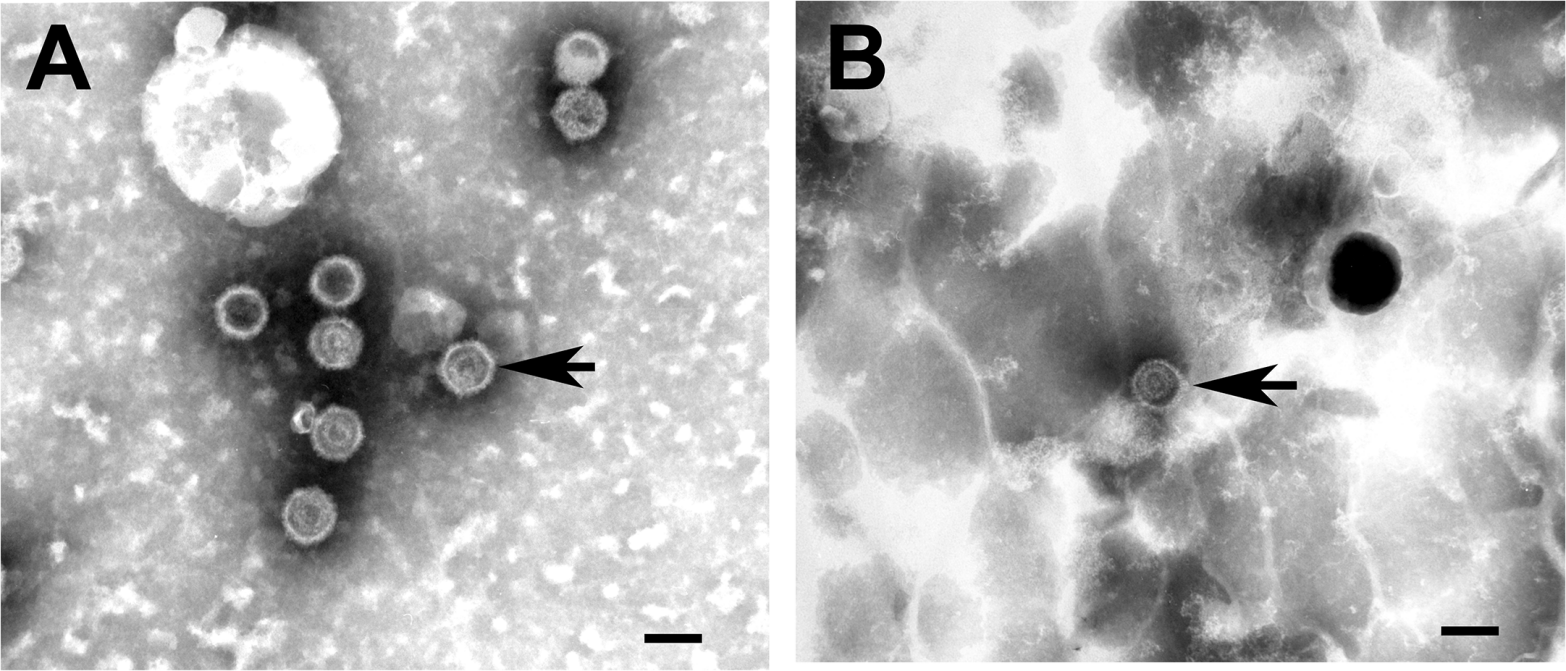
Electron micrographs of novel herpesviruses. Transmission electron microscopy was used to visualise herpesviruses in cultures of primary wombat kidney cells. Herpesvirus capsids (arrowheads) of VoHV-1 (A) and VoHV-2 (B) are shown. Bar = 100 nm.

Selected swabs that were PCR-positive for the presence of herpesviruses were used to inoculate either wallaby fibroblast cells or wombat kidney cells. Vombatid herpesvirus 1 and 2 and PhaHV-1 were successfully isolated on wombat kidney cells, MaHV-5 was successfully isolated on wallaby fibroblast cells. All the isolated viruses produced CPE characteristic of herpesvirus infection. The presence of herpesvirus virions was confirmed in the cultures using electron microscopy (Fig 2)

### Serological analysis

Serum samples from 79 animals from 8 different marsupial species were tested for antibodies against the two closely related macropodid alphaherpesviruses, MaHV-1 and MaHV-2. The seroprevalence of antibodies to MaHV-1 or -2 in the species evaluated ranged from 0.0–100%, with a pan-species prevalence of 44.3%. The results are summarised in Table 3.

**Table 3:** Seroprevalence of antibodies to MaHV-1 or MaHV-2 in serum samples collected from Australian marsupials in 2010 and 2011.

Family	Species	Number positive	Prevalence % (95% CI)
Macropodidae	Eastern grey kangaroo	23/25	92.0 (74.0–99.0)
	Swamp wallaby	1/1	100 (5.0–100.0)
	Brush-tailed rock wallaby	0/4	0.0 (0.0–52.7)
	Tammar wallaby	0/4	0.0 (0.0–52.7)
	Yellow-footed rock wallaby	0/1	0.0 (0.0–95.0)
Phascolarctidae	Koala	0/8	0.0 (0.0–31.2)
Dasyuridae	Tasmanian devil	1/21	4.8 (0.1–23.8)
Vombatidae	Common wombat	10/15	66.7 (38.4–88.2)
All families	All species	35/79	44.3 (33.1–55.9)

### Risk factors

The epidemiological variables of sex, age, body condition score, presence or absence of disease, season of sample collection, source (wild or captive) and the presence or absence of pouch young/lactation (in females only) were assessed as predictors for the presence of herpesvirus DNA in the collected swab samples in species in which 10 or more individual animals tested positive for the presence of herpesvirus DNA. *Chlamydia pecorum* status and geographical location were also assessed as variables for the sub-selection of koala samples (n = 68) for which this information was available [18]. Univariable analysis results for each of these species (eastern grey kangaroos, koalas, Tasmanian devils and common wombats) are shown in Tables 4–7. Statistically significant associations, as determined by multivariable analysis, are presented in Table 8. Similar analyses were performed for all marsupial species combined, including those species in which less than ten individual species tested positive for the presence of herpesvirus DNA. The results from this pan-species analysis are available as supplementary material (S1 and S2 Tables).

**Table 4:** Univariable analysis assessing select epidemiological variables as predictors for the presence of herpesvirus DNA in eastern grey kangaroos ^a^.

Variable	Herpesvirus DNA positive	Prevalence (%)	Odds ratio	95% CI	Wald p value	Log likelihood p value
**Sex**						**0.048**
Female	10/57	17.5	1.0			
Male	12/33	36.4	2.7	1.0–7.2	0.049	
Unknown	2/6					
**Age**						**0.13**
Pouch young/ sub-adult	12/35	34.3	2.1	0.8–5.5	0.13	
Adult/aged	11/55	20.0	1.0			
unknown	1/6					
**Wild/captive**						n/a
Wild	24/96	25.0	n/a	n/a	n/a	
Captive	0/0					
**Pouch young/lactation (females)**						**0.005**
No	8/23	34.8	8.5	1.6–45.2	0.012	
Yes	2/34	5.9	1.0			
**Body condition score**						**0.19**
≤ 2	12/36	33.3	1.9	0.7–5.3	0.20	
≥ 3	9/44	20.5	1.0			
Unknown	3/16					
**Disease present**						**0.048**
No	12/62	19.4	1.0			
Yes	12/31	38.7	2.6	1.0–6.9	0.048	
Unknown	0/3					
**Season**						**0.19**
Summer	0/2	0.0	0	n/a	0.99	
Autumn	3/5	60.0	5.7	0.8–38.9	0.076	
Winter	10/48	20.8	1.0			
Spring	8/26	30.8	1.7	0.6–5.0	0.34	
Unknown	3/15					

^a^ Reference levels are indicated by odds ratio of 1.0. Results highlighted in bold (log likelihood p ≤ 0.25) represent variables included in the initial multivariable model, with the exception of presence of pouch young/lactation as it is correlated with sex and thus excluded. Backward elimination of non-significant variables yielded no significant variables. Multivariable analysis was repeated including presence of pouch young/lactation as a variable instead of sex. Age was excluded from the model due to collinearity. In the final model (n = 42) only the absence of pouch young/lactation was identified as a significant factor (Table 8). n/a = not applicable.

**Table 5:** Univariable analysis assessing select epidemiological variables as predictors for the presence of herpesvirus DNA in koalas ^a^.

Variable	Herpesvirus DNA positive	Prevalence (%)	Odds ratio	95% CI	Wald p value	Log likelihood p value
**Sex**						**0.001**
Female	14/64	21.9	1.0			
Male	18/33	54.5	4.3	1.7–10.6	0.002	
Unknown	1/2					
**Age**						**0.056**
Sub-adult	1/13	7.7	0.15	0.02–1.2	0.079	
Adult	22/62	35.5	1.0			
Aged	7/16	43.8	1.4	0.5–4.3	0.54	
Unknown	3/8					
**Body condition score**						0.55
≤ 2	5/13	38.5	1.5	0.4–5.0	0.54	
≥ 3	20/67	29.9	1.0			
Unknown	8/19					
**Pouch young/lactation (females)**						0.55
No	12/49	24.5	1.0			
Yes	3/17	17.6	0.66	0.16–2.7	0.56	
**Disease present**						**0.024**
No	22/75	29.3	1.0			
Yes	10/17	58.8	3.4	1.1–10.2	0.026	
Unknown	1/7					
***C*. *pecorum* status**						**< 0.001**
Negative	3/48	6.3	1.0			
Positive	16/20	80.0	60.0	12.1–297.8	< 0.001	
Unknown	14/31					
**Wild/captive**						0.81
Wild	30/89	33.7	1.0			
Captive	3/10	30.0	0.84	0.2–3.5	0.81	
**Location**						**< 0.001**
Raymond Island	18/44	40.9	1.0			
French Island	1/24	4.2	0.06	0.01–0.51	0.009	
Other	14/31	45.2	1.2	0.5–3.0	0.71	
**Season**						**0.002**
Summer	0/1	0.0	0.0	n/a	0.99	
Autumn	21/50	42.0	6.3	1.7–23.5	0.006	
Winter	3/29	10.3	1.0			
Spring	2/2	100.0	n/a	n/a	0.99	
Unknown	7/17					

^a^ Reference levels are indicated by odds ratio of 1.0. Results highlighted in bold (log likelihood p ≤ 0.25) represent variables included in the initial multivariable model, with the exception of season as the timing of sampling correlated with the location at which it occurred and was thus excluded. In the final model (n = 68) only the presence of *Chlamydia pecorum* was identified as a significant factor (Table 8). n/a = not applicable.

**Table 6:** Univariable analysis assessing select epidemiological variables as predictors for the presence of herpesvirus DNA in Tasmanian devils ^a^.

Variable	Herpesvirus DNA positive	Prevalence (%)	Odds ratio	95% CI	Wald p value	Log likelihood p value
**Sex**						**0.25**
Female	9/32	28.1	1.0			
Male	8/18	44.4	2.0	0.6–6.8	0.25	
**Age**						**0.030**
Pouch young/ sub-adult	10/19	52.6	3.8	1.1–13.1	0.033	
Adult/aged	7/31	22.6	1.0			
**Wild/captive**						**0.001**
Wild	2/21	9.5	1.0			
Captive	15/29	51.7	10.2	2.0–51.9	0.005	
**Pouch young/lactation (females)**						0.69
No	5/16	31.3	1.0			
Yes	4/16	25	0.73	0.16–3.5	0.69	
**Body condition score**						0.63
≤ 2	3/7	42.9	1.5	0.3–7.7	0.63	
≥ 3	13/39	33.3	1.0			
Unknown	1/4					
**Disease present**						**0.13**
No	16/42	38.1	1.0			
Yes	1/8	12.5	0.23	0.03–2.1	0.19	
**Season**						**0.001**
Summer	15/29	51.7	10.2	2.0–51.9	0.005	
Autumn	0/0		n/a			
Winter	0/0		n/a			
Spring	2/21	9.5	1.0			

^a^ Reference levels are indicated by odds ratio of 1.0. Results highlighted in bold (log likelihood p ≤ 0.25) represent variables included in the initial multivariable model, with the exception of season as it was directly influenced by timing of management procedures, and therefore correlated with captive status and was thus excluded. In the final model (n = 50) only captivity was identified as a significant factor (Table 8). n/a = not applicable.

**Table 7:** Univariable analysis assessing select epidemiological variables as predictors for the presence of active herpesvirus infection in common wombats ^a^.

Variable	Herpesvirus DNA positive	Prevalence (%)	Odds ratio	95% CI	Wald p value	Log likelihood p value
**Sex**						1.0
Female	5/11	45.5	1.0	0.2–4.3	1.0	
Male	10/22	45.5	1.0			
**Age**						**0.006**
Pouch young/ sub-adult	3/15	20.0	1.0			
Adult/aged	12/18	66.7	8.0	1.6–39.6	0.011	
**Wild/captive**						n/a
Wild	15/33	45.5	n/a	n/a	n/a	
Captive	0/0					
**Pouch young/lactation (females)**						**0.095**
No	5/9	55.5	1.0			
Yes	0/2	0.0	0.0	n/a	0.99	
**Body condition score**						**0.060**
≤ 2	7/10	70.0	4.4	0.9–21.7	0.071	
≥ 3	8/23	34.8	1.0			
**Disease present**						0.95
No	9/20	45.0	1.0			
Yes	6/13	46.2	1.1	0.3–4.3	0.95	
**Season**						0.88
Summer	0/0					
Autumn	1/3	33.3	0.7	0.04–11.3	0.78	
Winter	3/7	42.9	1.0			
Spring	11/23	47.8	1.2	0.2–6.7	0.82	

^a^ Reference levels are indicated by odds ratio of 1.0. Results highlighted in bold (log likelihood p ≤0.25) represent variables included in the initial multivariable model, with the exception of pouch young which was excluded due to zero prevalence of herpesvirus infection in females lactating/with pouch young. In the final model (n = 33) only age (adult/aged) and body condition score (≤ 2) were identified as significant factors (Table 8). n/a = not applicable.

**Table 8:** Summary of epidemiological variables significantly associated with the presence of herpesvirus DNA in samples collected from different species of Australian marsupials in 2010 and 2011, as determined using multivariable analysis.

Population	Variable	Odds ratio (95% CI)	p value
Eastern grey kangaroos (n = 42)	Pouch young/lactation absent^a^	9.6 (1.3–72.5)	0.028
Koalas (n = 68)	*Chlamydia pecorum* positive	60.0 (12.1–297.8)	< 0.001
Tasmanian devils (n = 50)	Captivity	10.2 (2.0–51.9)	0.001
Common wombats (n = 33)	Adult or aged	17.4 (1.9–162.4)	0.012
	Poor body condition score (≤ 2)	11.7 (1.1–123.2)	0.041

^a^ The presence or absence of pouch young/lactation was assessed only in female animals.

## Discussion

This is the largest published study of herpesviruses in Australian marsupials to date, detecting DNA from 10 different herpesviruses in the samples tested. The findings demonstrate the common presence of herpesvirus infection in Australian marsupials and describe a number of important epidemiological associations. The study identified six novel herpesviruses across four marsupial species. Three of these novel herpesviruses were successfully isolated on primary wombat kidney cells or wallaby fibroblast cells. Herpesvirus DNA was detected from a number of different anatomical sites in the marsupial species tested, this information could be used to help inform sample collection strategies for future studies.

In this study three novel herpesviruses were detected in common wombats. This is the first time herpesvirus infections have been detected in common wombats by molecular techniques. It was found that adult and aged wombats had 17.4 times the odds of testing herpesvirus positive than juvenile wombats (p = 0.012). These data are consistent with an earlier serological study of Australian marsupials which found seropositivity increased with age [9]. The observed association between poor body condition and the detection of herpesvirus DNA in the common wombat (OR = 11.7, p = 0.041) may indicate the presence of disease caused by herpesvirus infection, or alternatively may be explained by a higher rate of new or reactivated herpesvirus infections as a consequence of immune-suppression associated with another disease process. The latter appears to be more likely in this study. With progressive urbanisation and habitat destruction in Australia, the generally solitary common wombat is increasingly being subjected to higher intraspecific competition for burrows and for food, with high rates of burrow sharing [23] and consequently higher stress levels and increased potential for disease transmission. Common wombats are particularly susceptible to infestation with the mite *Sarcoptes scabiei* var. *wombatii*. Sarcoptic mange is a major cause of debilitation, reduced reproduction and mortality in common wombats [24,25]. Such chronic debilitation could render infested animals more susceptible to new or reactivated herpesvirus infection. The discovery of herpesviruses (and especially alphaherpesvirus) infections in animals where sarcoptic mange is present is therefore of potential significance. The relatively high seroprevalence of antibodies to MaHV-1 and 2 in wombats in this study (66.7%) does not necessarily indicate infection with these viruses, but could indicate infection with another alphaherpesvirus (or multiple alphaherpesviruses) that cross-neutralise with MaHV-1 or -2, potentially VoHV-3. Future research directed at understanding the significance of infection with the three novel herpesviruses identified in this study is warranted. Expanding future studies to include the endangered northern hairy-nosed wombat (*Lasiorhinus kreffiti*) and vulnerable southern hairy-nosed wombat (*Lasiorhinus latifrons*) would also be informative.

In koalas, previous reports of herpesvirus infection (PhaHV-1 and PhaHV-2) have been limited to the description of individual cases [11,12]. In the current study, herpesvirus DNA was detected in 33.3% of the koalas at the time of sampling and there was a significant association between herpesvirus infection and concurrent infection with *C*. *pecorum* (OR = 60, p < 0.001). Infections with *Chlamydia* spp. have been extensively studied in koalas. *Chlamydia* is a significant cause of infertility and morbidity in wild koala populations, although infections can also be subclinical [18,26]. Coinfection of herpesviruses with members of the *Chlamydiaceae* family has also been observed in cats and humans [27–30], although co-infection appears to be less common in these species than in the koalas examined in this study. The association between *C*. *pecorum* and herpesvirus infection in koalas could represent concomitant transmission of both pathogens; reactivation of latent herpesvirus infection secondary to increased immunological burden associated with concurrent *Chlamydia* infection (or vice versa); or possibly a synergistic role in the pathogenesis of clinical disease in the koala. Further studies to investigate the relationship between herpesviruses and *Chlamydia* in koalas are required to determine the reasons for this association and the impacts that coinfection may have on koalas health.

Our study demonstrated a high prevalence of herpesvirus infections in eastern grey kangaroos. High levels of seropositivity to the alphaherpesviruses MaHV-1 and 2 have been reported in eastern grey kangaroos previously [4,5,9]. Interestingly, the only alphaherpesvirus detected in eastern grey kangaroos in this study was MaHV-4, which has only been recently described [8]. It is probable that the seropositivity to MaHV-1 and -2 detected in this study could reflect infection (including latent infection) with MaHV-4, given there is serological cross-reactivity between these macropodid alphaherpesviruses [8]. The gammaherpesvirus MaHV-3 was detected in 20% of eastern grey kangaroos in this study, but this virus was unlikely to contribute to the detected seropositivity, as antibodies against MaHV-3 are unlikely to cross-neutralise the alphaherpesviruses MaHV-1 or -2. In multivariable analysis, non-lactation/absence of pouch young in female animals was the only factor associated significantly with the presence of herpesvirus DNA in this species (OR = 9.6, p = 0.028). This is contrary to observations in other species where lactation has been associated with reactivation of latent herpesvirus infection [31,32].

A novel gammaherpesvirus (DaHV-2) was detected in 34% of Tasmanian devils evaluated in this study. Although the Tasmanian devil has been extensively studied in recent years in an attempt to understand the aetiology of devil facial tumour disease (DFTD) [33], herpesviruses have not been previously identified in this species. Epidemiologic analysis identified captivity as a significant risk factor for detection of DaHV-2, which echoes the findings of Webber and Whalley (1978) who identified a higher seroprevalence and higher titre level of anti-MaHV-1 antibodies in captive marsupials compared with free-living counterparts, although different species were represented in the two studies [9]. In general, disease outbreaks and mortality events as a result of marsupial herpesviruses have predominantly been reported in captive animals [3,6,15,16,34]. This trend is presumably due to the close proximity in which captive animals are housed allowing for increased rates of transmission, including transmission to marsupial species that are not natural hosts of the transmitted herpesviruses, and the potential for higher rates of stress in the captive animals. High levels of stress have been reported in wild-caught Tasmanian devils introduced to captivity [35], and such stress could potentially be associated with the reactivation of latent herpesvirus infection. Importantly, no difference was observed in the rates of herpesvirus infection between healthy Tasmanian devils and those with disease (principally DFTD), indicating that DaHV-2 is not directly associated with disease. Serum from one captive Tasmanian devil tested positive in our serum-virus neutralisation assays, indicating infection with MaHV-1 or MaHV-2, or another related alphaherpesvirus. This could be an undetected alphaherpesvirus adapted to Tasmanian devils, or an alphaherpesvirus acquired from another host. Macropodoids can form part of the diet of both captive and wild Tasmanian devils and so this is a potential mechanism of herpesvirus transmission.

The identification and isolation of a novel gammaherpesvirus (MaHV-5) in healthy swamp wallabies, and the identification of a novel gammaherpesvirus (PeHV-1) in a southern brown bandicoot, are interesting findings. Further research involving larger numbers of animals is required to establish the prevalence of these viruses and to determine their significance to their hosts. At the pan-species level a number of parameters were identified as positive predictors for the presence of herpesvirus DNA (S1 and S2), including male animals, animals sampled in summer months and animals in poor body conditions. Although the results obtained from analysis of data from multiple species should be interpreted with care due to the potential for confounding effects, this information could be useful for informing the design of future studies of herpesviruses in marsupial species where limited preliminary data is available.

This study is the first to demonstrate the widespread nature of herpesvirus infection in a range of Australian marsupial species. The relatively high prevalence of herpesviruses in a variety of marsupial species and the large number of different herpesviruses identified have implications for the management of captive animal collections and other programmes that relocate animals for the purpose of population management. Further systematic surveillance of wild and captive animal populations and the development of more marsupial specific reagents, such as cell lines for virus propagation, are integral to the further study of these viruses.

## Supporting Information

S1 Table Univariable analysis assessing select epidemiological variables as predictors for the presence of herpesvirus DNA in the study population of all marsupials. (DOCX)

S2 Table Summary of epidemiological variables significantly associated with the presence of herpesvirus DNA in samples collected in the study population of all marsupials, as determined using multivariable analysis. (DOCX)
