# Supplementary material for: Prevalence and Clinical Significance of Herpesvirus Infection in Populations of Australian Marsupials
Source: PLoS One. 2015 Jul 29;10(7):e0133807. doi: 10.1371/journal.pone.0133807 (PMC4519311; doi:10.1371/journal.pone.0133807)
Supplement: S1 Table — (DOCX) [file pone.0133807.s001.docx]

**Table S1. Univariable analysis assessing select epidemiological variables as predictors for the presence of herpesvirus DNA in the study population of all marsupials ^a^**

| **Variable** | **Herpesvirus DNA positive** | **Prevalence (%)** | **Odds ratio** | **95% CI** | **Wald p value** | **Log likelihood p value** |
| --- | --- | --- | --- | --- | --- | --- |
| **Sex** | | | | | | **< 0.001** |
| Female | 39/224 | 17.4 | 1.0 |  |  |  |
| Male | 56/158 | 35.4 | 2.6 | 1.6 - 4.2 | < 0.001 |  |
| Unknown | 3/15 |  |  |  |  |  |
| **Age** | | | | | | 0.34 |
| Pouch young/ sub-adult | 27/98 | 27.6 | 1.2 | 0.7 - 2.1 | 0.50 |  |
| Adult | 58/245 | 23.7 | 1.0 |  |  |  |
| Aged | 8/21 | 38.1 | 2.0 | 0.8 - 5.0 | 0.15 |  |
| Unknown | 5/33 |  |  |  |  |  |
| **Wild/captive** | | | | | | 0.36 |
| Wild | 80/311 | 25.97 | 1.0 |  |  |  |
| Captive | 18/86 | 20.9 | 0.76 | 0.43 - 1.4 | 0.36 |  |
| **Pouch young/lactation (females)** | | | | | | **0.007** |
| No | 29/124 | 23.4 | 1.0 |  |  |  |
| Yes | 10/100 | 10.0 | 0.36 | 0.17 - 0.79 | 0.011 |  |
| **Body condition score** | | | | | | **0.012** |
| ≤ 2 | 28/77 | 36.4 | 2.0 | 1.2 - 3.5 | 0.011 |  |
| ≥ 3 | 56/255 | 22.0 | 1.0 |  |  |  |
| Unknown | 14/65 |  |  |  |  |  |
| **Disease present** | | | | | | **0.028** |
| No | 67/294 | 22.8 | 1.0 |  |  |  |
| Yes | 29/83 | 34.9 | 1.8 | 1.1 - 3.1 | 0.026 |  |
| Unknown | 2/20 |  |  |  |  |  |
| **Season** | | | | | | **0.054** |
| Summer | 15/35 | 42.9 | 2.9 | 1.3 - 6.6 | 0.008 |  |
| Autumn | 27/94 | 28.7 | 1.6 | 0.8 – 3.0 | 0.15 |  |
| Winter | 25/123 | 20.3 | 1.0 |  |  |  |
| Spring | 19/84 | 22.6 | 1.2 | 0.6 - 2.3 | 0.69 |  |
| Unknown | 12/61 |  |  |  |  |  |

^a^ Reference levels are indicated by odds ratio of 1.0. Results highlighted in bold (p ≤ 0.25) were included in the initial multivariable analysis with the exception of the pouch young/lactating variable which is correlated with sex and thus excluded. In the final model (n = 320) only sex (male), season (summer) and body condition score (≤ 2) were identified as significant factors (see Table S2).
