# Supplementary material for: Prevalence and Clinical Significance of Herpesvirus Infection in Populations of Australian Marsupials
Source: PLoS One. 2015 Jul 29;10(7):e0133807. doi: 10.1371/journal.pone.0133807 (PMC4519311; doi:10.1371/journal.pone.0133807)
Supplement: S2 Table — (DOCX) [file pone.0133807.s002.docx]

**Table S2. Summary of epidemiological variables significantly associated with the presence of herpesvirus DNA in samples collected in the study population of all marsupials, as determined using multivariable analysis**

| **Population** | **Variable** | **Odds ratio**  **(95% CI)** | **p value** |
| --- | --- | --- | --- |
|  | Male | 3.2 (1.8 – 5.5) | < 0.001 |
| All marsupials (n = 320) | Poor body condition score (≤ 2) | 3.1 (1.7 – 5.7) | < 0.001 |
|  | Samples collected in summer^a^ | 4.5 (1.8 – 11.2) | 0.001 |

^a^ Winter was used as the reference group
